# Genome Editing in Bacteria: CRISPR-Cas and Beyond

**DOI:** 10.3390/microorganisms9040844

**Published:** 2021-04-15

**Authors:** Ruben D. Arroyo-Olarte, Ricardo Bravo Rodríguez, Edgar Morales-Ríos

**Affiliations:** Departamento de Bioquímica, Centro de Investigación y Estudios Avanzados del Instituto Politécnico Nacional (CINVESTAV), Mexico City 07360, Mexico; rubendao@gmail.com (R.D.A.-O.); richiebr@ciencias.unam.mx (R.B.R.)

**Keywords:** CRISPR-Cas, prokaryotes, genome editing, ribonucleoprotein, suicide plasmids

## Abstract

Genome editing in bacteria encompasses a wide array of laborious and multi-step methods such as suicide plasmids. The discovery and applications of clustered regularly interspaced short palindromic repeats (CRISPR)-Cas based technologies have revolutionized genome editing in eukaryotic organisms due to its simplicity and programmability. Nevertheless, this system has not been as widely favored for bacterial genome editing. In this review, we summarize the main approaches and difficulties associated with CRISPR-Cas-mediated genome editing in bacteria and present some alternatives to circumvent these issues, including CRISPR nickases, Cas12a, base editors, CRISPR-associated transposases, prime-editing, endogenous CRISPR systems, and the use of pre-made ribonucleoprotein complexes of Cas proteins and guide RNAs. Finally, we also address fluorescent-protein-based methods to evaluate the efficacy of CRISPR-based systems for genome editing in bacteria. CRISPR-Cas still holds promise as a generalized genome-editing tool in bacteria and is developing further optimization for an expanded application in these organisms. This review provides a rarely offered comprehensive view of genome editing. It also aims to familiarize the microbiology community with an ever-growing genome-editing toolbox for bacteria.

## 1. Introduction

**Figure 1:**
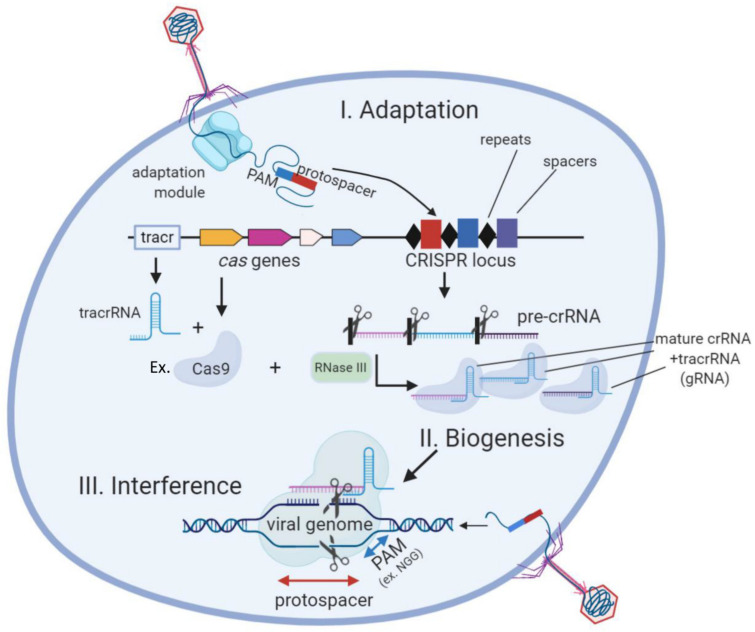
The three stages of the CRISPR-Cas (type II) bacterial adaptive immune system. During CRISPR adaptation, the injection of phage DNA into bacterial cell activates the adaptation module proteins which excise spacer-sized fragments of phage DNA for incorporation into CRISPR loci. During CRISPR RNA biogenesis, CRISPR loci are transcribed and resulting pre-crRNA is processed by a Cas9/RNaseIII complex at repeat sequences to generate mature crRNAs that couple to tracrRNA (gRNA). Individual gRNAs are bound by Cas protein effectors (e.g., Cas9). After a new phage infection with sequences matching a CRISPR spacer appears in the cell (lower right), specific Cas/gRNA complexes bind to viral DNA and cleave it.

Genome editing is the cornerstone for scientists to interrogate the genetic basis of physiological and metabolic processes in any organism, particularly in bacteria of scientific and industrial relevance. A series of classical genetic methods have been developed for bacterial species amenable to culture and transformation, e.g., suicide plasmids. These methods are highly laborious and usually, though not always (e.g., ClosTron method) require the introduction of at least one resistance marker cassette in the genome, which hampers the possibility of producing precise edits like single amino acid mutations [1]. In this regard, the current state-of-the art approach for genome editing in bacteria is to combine homologous recombination of a DNA template with DNA targeting by programmable nucleases from CRISPR-Cas systems [2]. These systems however have not yet been applied as widely as in eukaryotes and different strategies need to be optimized depending on the host species. Here, we discuss about the different strategies for genome edition in bacteria compared to CRISPR-Cas and also the most recent advances in this technology for these organisms. CRISPR (Clustered Regularly Interspaced Short Palindromic Repeats) is the only known adaptive and hereditary immune response in prokaryotes. It is present in about 50% of bacteria and 90% of archaea [3]. CRISPR-Cas acts by recompiling and storing genetic sequences from invader bacteriophages and noxious plasmids as spacers. These spacers are transcribed into crRNAs that bind to effector CRISPR nucleases (Cas proteins), which target specific complementary sequences, given they fulfill a specific PAM sequence requirement [4,5]. It was previously discovered that crRNAs need to couple to a RNAse III-edited tracRNA before binding to the Cas nuclease [6] (Figure 1). Depending on the number of effector proteins there are several types (I to VI) of CRISPR systems. Type II systems (e.g., Cas9) only depend on one effector nuclease which facilitates its heterologous expression, and are therefore the most popular tools for genome editing. 

## 2. Methods for Genome Editing in Prokaryotes

There are several methods that have been developed for genome editing in bacteria and are still widely used besides CRISPR-based tools. These methods, however, are highly laborious, often show inconsistent efficiencies, and require extensive tailoring for programming compared to simple gRNA design for CRISPR. Some of the most representatives are:

### 2.1. Suicide Plasmids

**Figure 2:**
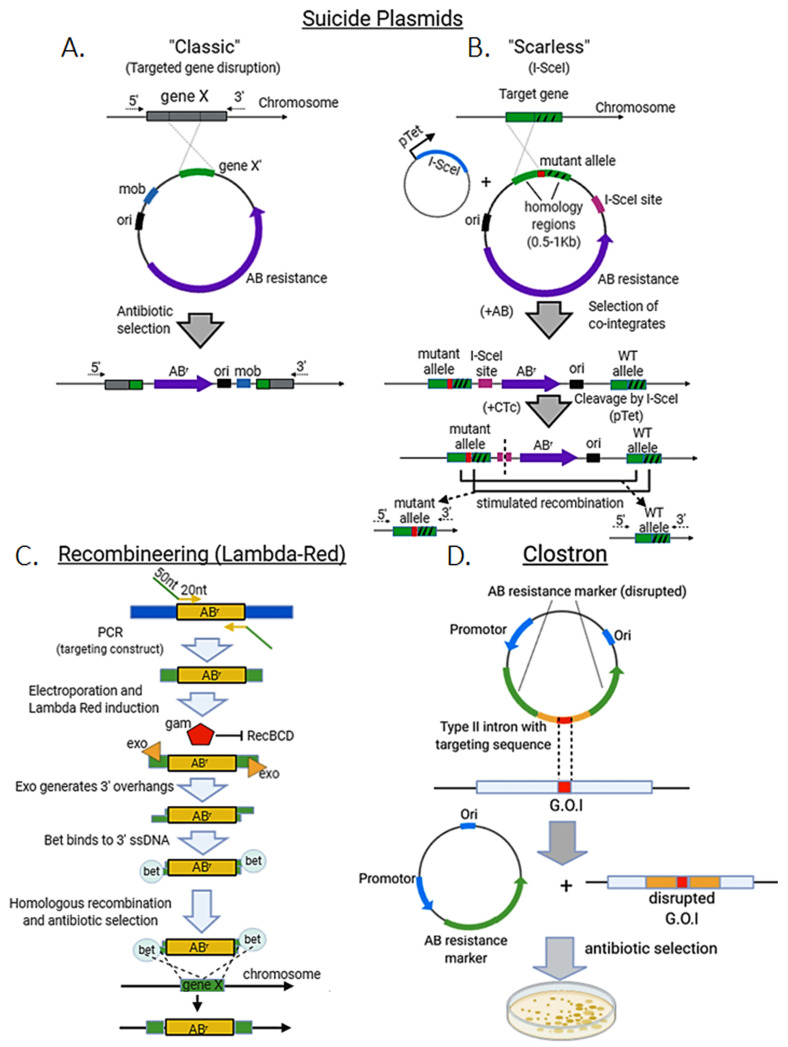
Standard methods for genome editing in bacteria. Suicide plasmids. (**A**) The classic approach consists in transforming with a non-replicating plasmid (usually with a transposon element, e.g., mob), which harbors a mutated recombination template and an antibiotic resistance marker (ABr). Antibiotic treatment will select only colonies that undergo homologous recombination to incorporate the plasmid sequence (including the mutant gene) at the target locus (disrupting gene X). (**B**) In the “scarless” variant a SceI site is incorporated in the plasmid to be transformed in a I-SceI expressing strain under an inducible promoter (pTet). After a first round of antibiotic treatment, cointegrating colonies harboring the plasmid sequence and the wild-type allele at the target gene locus are selected. Addition of chlortetracycline (CTc) induces I-SceI expression to cleave the target locus, which enhances homologous recombination to eliminate plasmid sequence resulting in either, reversion to wild-type or fixation of the mutant allele. (**C**) Recombineering (lambda red system) for targeted gene disruption. A targeting construct with 50 nt of homologous sequence at the 5′ and 3′ ends and antibiotic resistance marker is made by PCR. PCR template is electroporated and expression of the lambda Red proteins is induced (Ex. Heat shock at 42 °C). Gam inhibits RecBCD nuclease activity upon linear DNA (protecting the targeting construct). Exo generates 3′ overhangs in the DNA linear template, which are accessed by bet protein to facilitate homologous recombination and integration and disruption of the target gene (gene x). Edited colonies are then selected by antibiotic treatment. (**D**) ClosTron method. A type II intron with transposon activity is cloned within a disrupted antibiotic resistance cassette in a plasmid. After transformation, the intron, which has been modified with a specific, homologous sequence, targets the gene of interest (G.O.I) and disrupts it leaving behind a plasmid with a functional antibiotic resistance marker. Antibiotic selection then enhances and simplifies the obtention of mutant colonies.

The first method developed were “suicide” plasmids in the 1980s [7]. Suicide plasmids are those that can replicate in one organism, but not in another called recipient. These plasmids contain a homologous sequence (with the desired insertion, deletion or site-directed mutation) coupled to a marker, usually an antibiotic resistance cassette, and may harbor a transposon sequence that facilitates their insertion into the genome of the recipient strain after conjugation with the donor. As plasmid replication is not possible in the recipient strain or species, antibiotic treatment will select only those colonies that undergo genome integration (“Classical” method, Figure 2A). This strategy however, usually has very low efficiency and can have a high rate of false positives, often requiring two rounds of selection with different antibiotics to achieve edited colonies [8,9]. It is still used in *E. coli*, but particularly in other prokaryotic organisms where novel alternatives such as CRISPR-Cas work poorly for large gene deletions/insertions, e.g., *Corynebacterium glutamicum* [9,10]. Moreover, site-directed mutants generated by this strategy have been useful for protein purification studies, e.g., a C-terminal truncated, soluble cytochrome c1 in *Rhodobacter sphaeroides* [11]. In this case, water-soluble domains of membrane-associated subunits of respiratory complexes usually are more amenable than the native proteins to crystallization for structural studies [12]. 

I-SceI is a homing endonuclease from *Saccharomyces cerevisiae* that targets an 18 bp asymmetric sequence (TAGGGATAACAGGGTAAT), cleaving both DNA strands to leave 3′-overhangs with a four base-pairs length, which can induce homologous recombination [13,14]. Suicide plasmids can incorporate an I-SceI site between the mutant allele and the antibiotic resistance marker. The suicide plasmid is transformed into an *E. coli* strain already harboring an inducible plasmid for I-SceI expression. The suicide plasmid is then integrated into the genome and colonies are selected by their antibiotic resistance at the non-permissive temperature for plasmid replication. Induction of I-SceI cleaves the target gene locus, which is then repaired via native RecA-mediated homologous recombination, providing large enough homology arms (>500 bp). This can result either in a reversion to the wild-type chromosome or in a markerless allele replacement [15] (“Scarless” method, Figure 2B). Theoretically, a 50:50 ratio between wild-type and mutant-allele colonies is expected; however, this will depend on the nature of the mutation. Small non-deleterious mutations are preferred over large deletions which cannot be repaired as efficiently [16]. In the latter case, as well as for those mutations leading to an even small growth defect, a large number of colonies need to be screened.

### 2.2. Lambda Red System

Another widely used approach for genome editing in prokaryotes is the lambda Red system [17]. This system is derived from the lambda bacteriophage and it is also known as “recombineering” (recombination-mediated genetic engineering) [1]. Lambda Red consists primarily of three proteins: α, β, and γ. α is an exonuclease (exo), which processively digests the 5’-ended strand of a dsDNA end. β (bet) binds to ssDNA and promotes strand annealing. Finally, γ (gam) binds to the bacterial RecBCD enzyme (which degrades any linear DNA used as a template) and inhibits its activities. These proteins induce a “hyper-recombination” state in *E. coli* and other bacteria, in which recombination events between DNA species with as little as 35–50 bp of shared sequence occur at high frequency [1,17,18]. The system itself is however selection-free and therefore is usually combined with the insertion of large antibiotic-resistance cassettes to improve the recovery of edited colonies [1] (Figure 2C). In some cases, I-SceI sites are also included in the targeting construct to proceed with a counter-selection step to eliminate the resistance marker by homologous recombination [19]. 

### 2.3. ClosTron Method

For decades genome editing in clostridia was hampered by the lack of mutational tools for functional genomic studies. The ClosTron method utilizes an endogenous intron with transposon activity, a bacterial group II intron, as an insertional gene inactivation tool [20,21]. These are broad host-range elements whose target specificity is determined largely by homology between intron RNA and target site DNA. Such introns can therefore be re-targeted by altering the sequence of an intron RNA-encoding plasmid. The ClosTron system uses an element derived from the broad host range Ll.LtrB intron of *Lactococcus lactis*. Intron target specificity is determined by a small region, so it is cost-effective to re-target an intron by sub-cloning of a small DNA fragment (Figure 2D).

In general, standard genome-editing methods still lack the simplicity and programmability of CRISPR-Cas, that would be crucial for more complex endeavors than single gene knockouts (e.g., genome-wide screenings).

## 3. CRISPR-Cas9 as a Genome-Editing Tool

Among the different Cas systems, the Cas9 protein from *Streptococcus pyogenes* (SpCas9) is currently the most widely used as a gene-editing tool. This is mostly due to its relatively common PAM sequence requirement: NGG (where N can be any nucleotide), with a theoretical frequency of once in every 8 bp in a random double-strand DNA sequence. The actual frequency of the PAM motif will vary across genomes and is expected to be much rarer in AT rich genomes. As an example, NGG has been calculated to occur approximately once every 42 bases in the human genome [22]. Recently, novel Cas9 variants with more relaxed or nearly absent PAM requirements have been developed, expanding the target site recognition of CRISPR-Cas9 [23]. The crRNA and tracrRNA can also be fused into a single-guide RNA molecule (sgRNA) with the same activity. The other key element of the CRISPR-Cas system is the recombination template that contains flanking homology arms, the desired edit (insertion, deletion or specific mutation), and an internal sequence that disrupts the target site (e.g., mutations to the PAM), preventing targeting upon successful recombination. Cleavage of unedited target genes by CRISPR nucleases is often lethal in bacteria because of the formation of a double-strand break (DSB), serving as a strong counterselection without the need of the insertion of a large resistance cassette marker into the genome. In this way the DSB drives editing through homologous recombination (HR) or, more rarely in bacteria, via non-homologous end joining (NHEJ) [24]. It is, therefore, the DNA repair systems of the host species/strain which actually perform the desired editing. In most bacterial organisms RecA-mediated HR is induced to repair DNA damage by DSB. This response however, is usually error-prone and inserts undesired mutations, mainly through the recruitment of the mutagenic DNA polymerase IV (PolIV) and inhibition of high-fidelity PolIII at the DSB site [25,26,27]. In most cases where CRISPR nucleases have been used to achieve highly efficient genome editing, particularly in *E. coli*, they are combined with an enhanced recombination system e.g., the Lambda Red phage to promote homology-directed repair (HDR) [28].

## 4. CRISPR-Cas9-Based Methods for Genome Editing in Bacteria

**Figure 3:**
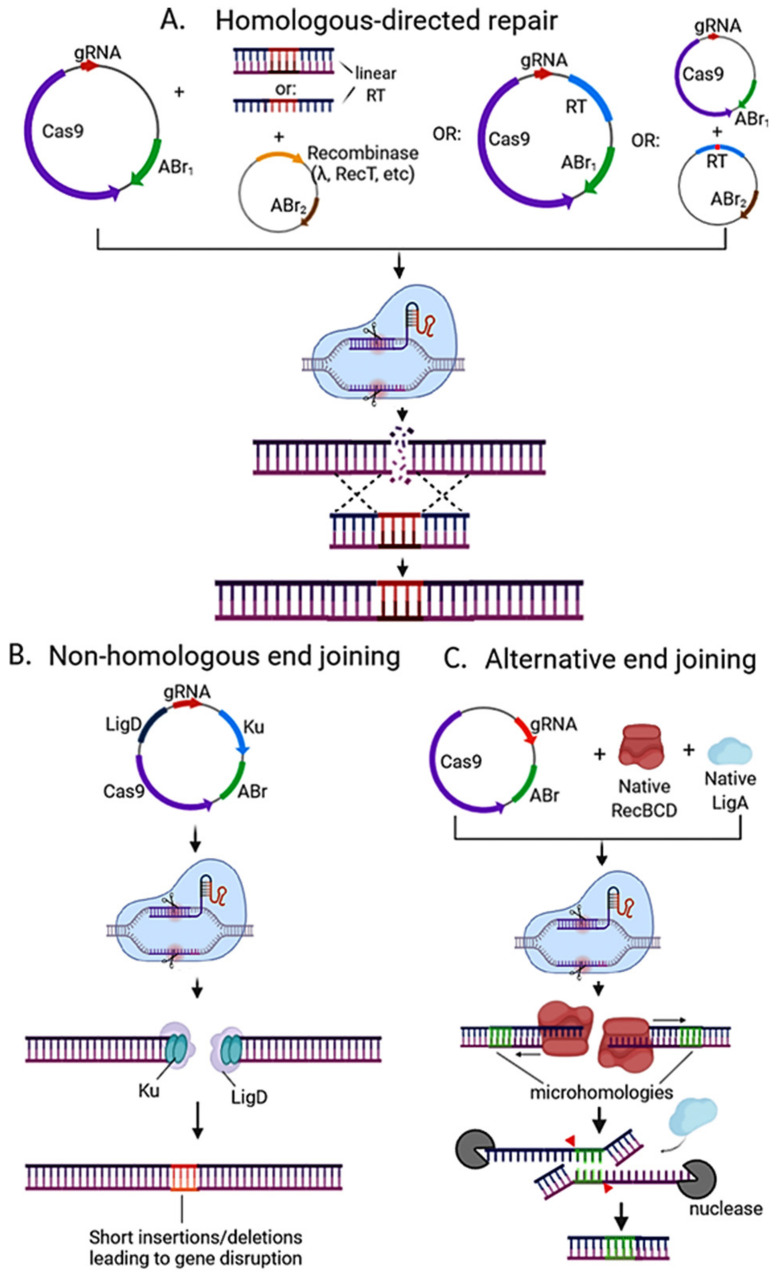
Strategies used for CRISPR-Cas based genome editing in bacteria. (**A**) Editing via homologous recombination: Recombineering with a linear DNA template is followed by counterselection with CRISPR nucleases. A heterologous recombinase (e.g., λ red, RecT) is introduced via a plasmid (or phage) into the cell and co-transformed with the linear DNA template and CRISPR-nuclease plasmid with respective antibiotic-resistance marker (ABr). Genome editing may also be directed with a plasmid-encoded recombination template (RT) and endogenous or heterologous recombinase. The recombination template can be placed on the same plasmid encoding the CRISPR machinery for an all-in-one plasmid system, or it can be placed on a separate plasmid before transforming the CRISPR nuclease/gRNA plasmid. One-plasmid system is more streamlined, but due to its larger size it can be hard to transform, and cloning may not be possible if the gRNA can target the genome of the cloning strain. (**B**) Editing via the non-homologous end-joining (NHEJ) pathway. Depending on the strain, ku and/or ligD can be encoded on the CRISPR nuclease/gRNA plasmid and transformed into the strain. (**C**) Alternative end joining (A-EJ) pathway can be found natively in many bacterial species with incomplete NHEJ. It does not require the introduction of foreign Ku or LigD, and instead relies in microhomology-directed repair via RecBCD, nucleases, and LigA, leading to deletions of variable sizes (depending on the location of microhomologies) at the Cas9 cut site. For a more detailed insight on NHEJ and A-EJ mechanisms, the reader is advised to read [45]. All strategies require plasmid curing after nuclease targeting to isolate the mutant strain in order to avoid interference in pursuing downstream applications.

Since its discovery, CRISPR Cas9 evolved as one of the main genome-editing tools in many organisms, including bacteria and a wide array of CRISPR-Cas9-based methods have been developed. These methods can vary on the number of plasmids used, the use of heterologous recombinase (e.g., lambda Red), and the DNA repair mechanism induced (e.g., HDR) (Figure 3).

The most common strategy used in *E. coli* and other model bacteria, uses a DNA linear template as well as a phage-derived recombinase to repair the DSB. In this approach (Figure 3A), the first step is to include and induce the expression of the foreign recombinase in a plasmid followed by co-transformation with the recombination DNA template and the CRISPR plasmid (Cas9+gRNA). The bottleneck here is the availability of a highly efficient recombinase to counter-select enough viable gene-edited colonies from DSB-killed non-edited colonies. In this regard, the original description of the use of SpCas9 for genome editing in *E. coli* used the lambda Red phage recombinase system and linear double-stranded DNA as template to incorporate the desired edits [28]. This system has been used in other species, mainly Proteobacteria [29,30,31,32,33]. Other heterologous recombination systems have recently been screened for their activity in different bacteria either alone or coupled to CRISPR-Cas. Among them, recombinase T (RecT), has been established successfully to enhance the CRISPR-Cas-mediated genome editing in *Corynebacterium glutamicum* [34], *Lactoccocus lactis* [35], *Lactobacillus plantarum*, and *Lactobacillus brevis* [36]. RecT binds to ssDNA and protects it from degradation, fulfilling a similar function to gamma protein in the lambda Red system [37].

Alternatively, the DNA repair template may be encoded in the same or different plasmid than SpCas9. In this case, foreign recombinases have been used [38,39], though native recombination machinery may also be relied upon (Figure 3A). This has been shown in *E. coli* with 1 Kb homology arms in the recombination template plasmid [40]. In their work, Vento et al. [2] described other bacteria where the native recombination machinery has been applied successfully with this approach, such as *Clostridium ljungdahlii* [41], *Lactobacillus plantarum* [42], *Pseudomonas putida* [43], *Streptomyces coelicolor* [44], and *Staphylococcus aureus* [45]. Using the native recombination machinery can simplify the system; however, in many species this machinery is either not reliable or efficient enough to achieve the desired edit. 

Recombination template and/or machinery may also be omitted when relying on the non-homologous end-joining pathway to repair the CRISPR-Cas-directed double-strand break (Figure 3B). However, very few bacterial species harbor a sufficiently active NHEJ machinery natively, therefore it must be usually heterologously encoded in the CRISPR plasmid. The NHEJ machinery in bacteria consists basically of two proteins: Ku and LigD. Ku binds to the cleaved DNA ends, while LigD joins them to seal the DNA together, often introducing non-specific mutations, insertions, or deletions that render the gene non-functional (Figure 3B). Similarly, the native alternative end-joining (A-EJ) pathway (also known as microhomology-mediated joining) can be exploited (Figure 3C). This DNA repair pathway relies on microhomologies (1–9 nt) near the cut site by Cas9, which after resection of DNA ends by RecBCD being ligated by LigA, leaving behind deletions of variable sizes after repair [46]. Native A-EJ has been combined with CRISPR-Cas9 in several species, including *E. coli* [47], *Streptomyces coelicolor* [48], and *Pectobacterium atrosepticum* [49]. Both strategies would not be useful to introduce specific mutations or insertions but would be effective for gene knockouts. 

Overall, these strategies are not mutually exclusive and may be combined depending on the host species. In any case, they may have common drawbacks related to the continuous expression of a foreign Cas9 protein. SpCas9 overexpression can be highly cytotoxic in *E. coli* and many other bacteria (it will be explained within the next section) leading to little or no colonies, even when devoid of its nuclease activity [50,51]. 

## 5. Alternatives to SpCas9-Associated Cytotoxicity and Lack of Colonies: Expanding the Toolbox

**Figure 4:**
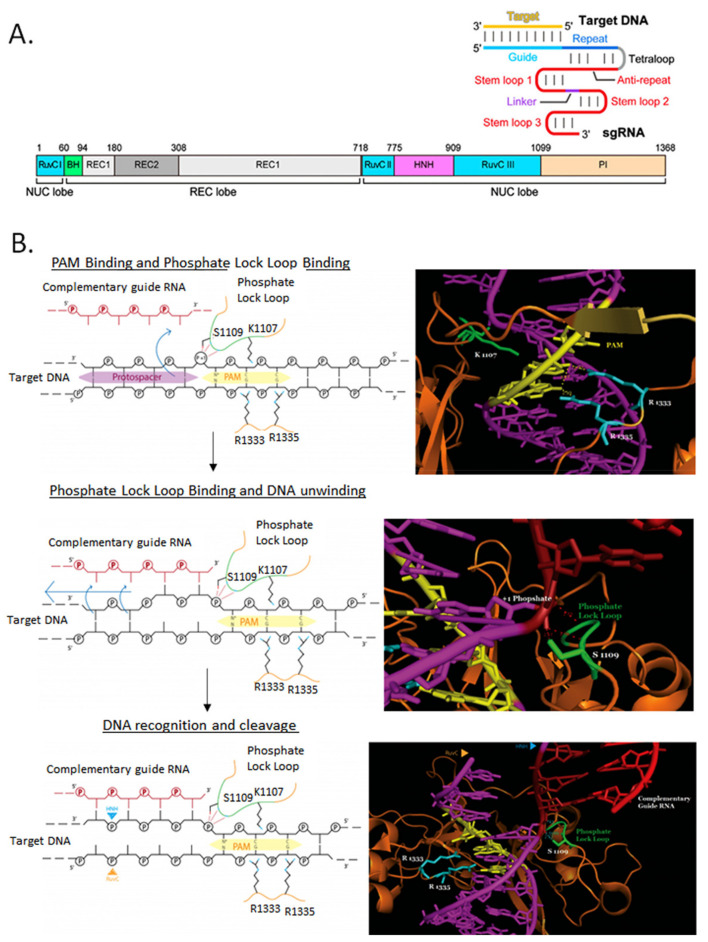
Molecular mechanism of SpCas9/gRNA cleavage of target DNA. (**A**) The main domains of Cas9 are illustrated next to a gRNA/target DNA secondary structure scheme. Adapted from [52]. (**B**) The first step is the PAM binding and phosphate lock loop binding, followed by DNA unwinding and finally the DNA recognition by gRNA and the target DNA cleavage by the RuvC and HNH nuclease domains at both strands. Critical Cas9 residues for each step are illustrated. Adapted from [54].

SpCas9 has been used almost exclusively to perform genome editing in bacteria since its original application in *E. coli* [28]. This is mostly due to its relatively simple PAM sequence requirement, but also to its well-characterized crystal structure and molecular mechanism of action (Figure 4). SpCas9 displays a striking conformational change upon gRNA binding. This in turn, uncovers two endonuclease domains, RuvC cleaving the non-target DNA strand while the HNH cleaves the target DNA strand complementary to the gRNA [52]. Another important aspect of SpCas9 mechanism is the recognition of the PAM sequence (NGG). The critical residues of the PAM-binding domains (Toro and CTD) involved in the hydrogen bonding to the dinucleotide GG of the PAM sequence are R1333 and R1335. This study, [52] highlights the central importance of PAM recognition in Cas9 function, both as a critical determinant of initial target DNA binding and as a required element in subsequent strand separation and gRNA-target DNA hybridization. Interestingly, these steps can tolerate up to 5 base-pair mismatches between the target DNA and gRNA sequence depending on their position and distribution [53]. Mismatches occurring in the PAM-proximal region, are usually less tolerated whether these mismatches are concatenated or interspaced; this effect is further magnified for three concatenated mismatches. In the PAM distal regions more than three interspaced, or five or more concatenated mismatches have been shown to eliminate any detectable SpCas9 cleavage in most human loci [53]. Cas9 mismatch-tolerance facilitates catalysis in certain situations (e.g., for polymorphic loci), but potentially also triggers double-strand breaks at off-target genome locations. Following general gRNA design guidelines, combined with the use bioinformatic tools to predict mismatches in a given target genome can minimize these effects. 

**Figure 5:**
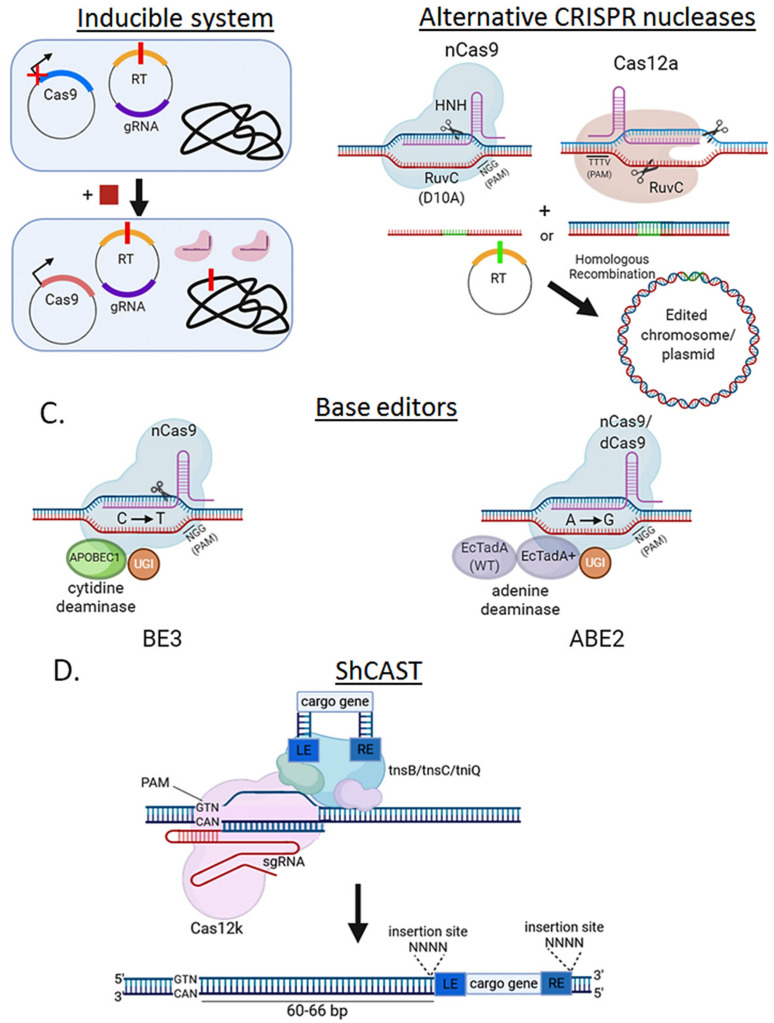
Alternative strategies to circumvent SpCas9 cytotoxicity. (**A**) Use of inducible systems to express SpCas9. Via an inducible promoter, SpCas9 expression is strongly repressed without inducer present (square) and only induced after exponential culture so that enough cells can survive and perform the genome edit. (**B**) Using less toxic nucleases to achieve editing. nCas9, which only cleaves one strand of DNA, and Cas12a (PAM: TTTV, where V is A or C or G) can be less toxic than SpCas9. (**C**) SpCas9-derived base editors eliminate the double-stranded break requirement for genome editing. A translational fusion of nCas9 (nickase) or dCas9 (“dead”), a cytidine (e.g., APOBEC1 in BE3) or adenosine (e.g., TadA-EcTadA+ in ABE2) deaminase domain, and an uracil DNA glycosylase inhibitor (UGI) is introduced on a plasmid into the cell. Upon nuclease binding and DNA strand unwinding, cytidines (or adenines) on the non-target strand within a defined window adjacent to the PAM are rapidly converted to uracil (or inosines), which is then processed as thymidine (or guanines) by DNA polymerase. (**D**) ShCAST insertion mechanism. A Tn7-like transposon from *Scytonema hoffmani* encodes transposases (tnsB, tnsC, tniQ), a nuclease deficient type V CRISPR protein (Cas12k) and guide RNA. This complex is combined with a cargo gene flanked by LE and RE elements. ShCAST is directed to the target locus and integrates the cargo gene 60–66 bp downstream of the PAM sequence, generating and insertion of the cargo gene flanked by the SE and RE elements, and a duplicated (4 bp) insertion site.

As mentioned earlier, overexpression of SpCas9 can be cytotoxic, potentially hindering any genome editing application. In certain species like *Corynebacterium glutamicum*, it is not possible to achieve the transformation of a SpCas9-encoding plasmid, even in the absence of gRNA, due to an absolute lack of colonies [10]. Initially, it was thought that SpCas9 cytotoxicity was due solely to residual, unspecific nuclease activity. One study showed that overexpression of a nuclease-devoid SpCas9 (dCas9) leads to abnormal morphology and reduced colonies, suggesting instead a role for its PAM recognition and DNA-binding activity across the genome [50]. This study also showed critical effects on cell division as well as inner and outer membrane structure, particularly in the absence of gRNA. On the other hand, in cases where SpCas9 expression can be well tolerated, genome targeting greatly reduces the cell survival even in the presence of a recombination template [40,55]. Therefore, in bacteria where there is also a poor transformation efficiency and/or weak DNA repair mechanisms, these effects sum up and can turn into no colonies when the system is used for genome editing. To ameliorate these issues, inducible promoters have been used to drive SpCas9 expression (Figure 5A). An IPTG-inducible promoter has been used in a single-plasmid CRISPR system for metabolic engineering through genome editing in *E. coli* [56]. Other available inducible promoters used to drive SpCas9 and/or gRNA expression are those dependent on tetracycline derivatives (pTet), mannose, nisin, and arabinnose in several bacteria including *E. coli* [57], *Bacillus subtilis* [58], *Clostridium acetobutylicum* [59], and *Lactococcus lactis* [60]. However, even under the control of these promoters, leaky SpCas9 expression at its “off” state has been shown to elicit significant background activity [61]. Light-inducible systems have been developed successfully in eukaryotic cells with little or no background SpCas9 activity [62,63], however they require specialized optical instruments and need yet to be tested in bacteria. 

SpCas9-RuvC domain has been mutagenized (D10A) to function as a DNA nickase to produce single-strand breaks instead of the more lethal DSB (Figure 5B). The resulting nicking SpCas9 (nCas9) has been shown to be useful as a genome editing tool in cases where transformation with SpCas9 plasmids leads to no colonies, especially for large-scale genome deletions [64]. However, because of the non-lethal nature of single-strand breaks, nCas9 cannot be used as a counter-selection tool, which usually leads to poor efficient genome editing [65,66]. Alternatively, two adjacent gRNAs targeting opposing DNA strands can be used together with nCas9 to generate a staggered double-strand break [67]. This last approach would be more specific and less prone to off-target edits, but also increases the PAM requirement (must be in both strands and in close proximity) and requires finding two gRNAs with high activity targeting a reduced stretch of base pairs. In *Corynebacterium glutamicum*, where neither exogenous SpCas9 nor nCas9 expression is possible because of the toxicity and consequent plasmid loss, SpCas9 gene has been introduced into the genome under a native promoter. The resulting strain showed a low rate of escape colonies and a high gene-editing efficiency when transformed with a plasmid encoding a specific gRNA and the recombination template [68].

Natural double-nicking CRISPR nucleases like the Type V-A Cas12a (also known as Cpf1) from *Francisella novicida* (FnCas12a) have also been characterized. Cas12a orthologs require one single gRNA and are usually smaller than SpCas9, recognizing a T-rich PAM and introduce a 5′ 5-nt overhang upon DNA cleavage [69] (Figure 5B). As an alternative to Cas9, in *Corynebacterium glutamicum*, a FnCas12a-encoding plasmid could be successfully transformed and used for genome editing [10]. Cas12a has since then been applied for genome editing in other bacteria, e.g., *Yersinia pestis* and *Mycobacterium smegmatis* [30]. There are, however, some aspects of this CRISPR nuclease that need to be characterized and further studied regarding its effects on genome editing. It has been found that once the Cas12a/gRNA complex cleaves its target DNA sequence, it remains active (contrary to Cas9, which is a single-turnover enzyme) and targets non-related sequences for cleavage. Although potentially troublesome for genome editing (possible off-targets), this feature has recently been applied for pathogen-infection diagnostics (e.g., SARS-Cov2) by cleaving fluorogenic DNA probes [70].

One of the most recent advances in genome editing are the base editors, which specifically perform single-nucleotide edits without a double strand break or recombination template. The most extended base editor, BE3, is composed of a chimera of nCas9 to provide strong, specific gRNA-programmable DNA binding, and cytidine-deaminases, e.g., APOBEC1, to conduct C to T editing in the target gene [71] (Figure 5C). Other variants of the system include an adenine-deaminase (A to G conversion) instead of a cytidine-deaminase [72]. Cas12a [73] or dCas9 [72] may also be used instead of nCas9. The advantages of these systems include its relative innocuity compared to Cas9-induced DSBs, and its independence from recombination machinery to introduce specific single-nucleotide mutations in a target gene. This system was initially developed in eukaryotes but is becoming more common in some bacteria like *E. coli* [74], *Klebsiella pneumonia* [32], *Pseudomonas aeruginosa* [75], *Rhodobacter sphaeroides* [76], and *Staphylococcus aureus* [77]. Recently, however, a transcriptome-wide off-target RNA editing activity has been shown to be triggered by continuous expression of base editors, particularly those based on cytidine-deaminases in mammalian and plant cells [78]. Similarly, embryonic cells expressing base-editors show a higher than normal frequency of single-nucleotide polymorphisms [79]. These reports are consistent with the fact that cytidine-deaminases like APOBEC1 and APOBEC3G have a well-documented anti-DNA and anti-RNA virus replication activity, mainly through hypermutating viral genomes [80,81,82,83]. Despite the encouraging results using base-editors in bacteria, more research is needed to address these possible caveats in prokaryotes. 

Although base-editors are optimized for single-base edits, replacing larger stretches of genomic DNA by inserting sequences such as an epitope tag or a deletion usually requires a foreign DNA donor to repair a Cas9-induced DSB. A type V-K CRISPR-associated transposase (ShCAST) system avoids these requirements (Figure 5D). This method is based on a naturally occurring Tn7-like transposon from *Scytonema hoffmani* which encodes besides its transposase genes, a nuclease-deficient Cas12k, tracRNA and 28–34 bp crRNAs [84]. ShCAST transposases, Cas12k and targeting sgRNAs are cloned into a helper plasmid, while cargo genes flanked by LE and RE elements to facilitate their insertion into a crRNA-targeted locus, are cloned into a donor plasmid. Integration is not “scarless” as it also includes the LE and RE elements and a 5-bp duplication at the insertion site. The ShCAST system has shown up to 80% genome editing efficiency in several *E. coli* target loci without positive selection, highlighting its potential for genome engineering in prokaryotes [84]. A similar approach has been demonstrated in *E. coli* using the CAST locus from *Vibrio cholerae* [85]. Prime-editing is another recent DNA repair-free editing method. It combines an nCas9 and a reverse transcriptase that utilizes a pegRNA that works as both a guide RNA and as a reverse-transcriptase template to generate a desired DNA sequence that is integrated in the target locus [86]. Prime editing shows higher or similar efficiency and fewer byproducts than homology-directed repair and induces much lower off-target editing than Cas9 nuclease at known Cas9 off-target sites in human cells [86]. Prime-editing has been successfully applied in mice [87] and plants [88], but its feasibility for bacterial genome editing still needs to be explored. Particularly, the large size of the prime-editing complex (about 7000 bp), may affect an efficient transformation and/or expression in bacteria. 

A more laborious but hopefully much less deleterious way to use CRISPR-based genomic editing is to harness the endogenous CRISPR systems of bacteria. This would require however an extensive characterization of CRISPR loci and endogenous CRISPR nucleases for each species. In this regard, it was recently demonstrated that the endogenous Cas9 of *Mycoplasma gallisepticum* (MgaCas9) is active and can be used to perform genome editing in this species with low dependency on adjacent sequences [89]. There are two major classes of CRISPR systems depending on the composition of effector genes involved, which are subdivided in six different types. Types I, III, and IV belong to Class 1 and require the activation of a Cascade-like complex that recognizes and cleaves the target as DNA nicking systems [90]. Type III systems in particular lack a PAM requirement and some of them (e.g., subtype III-B) target RNA [91]. On the other hand, class 2 systems (type II, type V, and type VI) need only one protein, to scan, bind, and cleave the target DNA or RNA sequence. Type II (e.g., Cas9) and V (e.g., Cas12) are the most commonly used for genome editing, while type VI (e.g., Cas13) are employed for RNA editing [92]. Despite being the most abundant CRISPR systems in prokaryotes, type I systems have not been used as often as type II and V systems for genome engineering, owing to the relative difficulty of heterologous expression of the multicomponent Cascade complex (Cas1-2, Cas5-8, Cas11, and Cas3 as final endonuclease effector). Endogenous CRISPR type I systems would obviate this requirement. In *Clostridium difficile*, an endogenous CRISPR type I system has been characterized and redirected for Cas3-driven, DSB-induced auto-immunity control of this human pathogen [93]. Another endogenous type I-A CRISPR system has also been exploited to facilitate genome editing by double-homologous recombination in *Heliobacterium modesticaldum* [94]. Interestingly, Cas3 from *Pseudomonas aeruginosa* has been repurposed not only as an endogenous genome-editing tool, but also as a heterologous editing tool more efficient than Cas9 for large deletions in *E. coli* and in the plant pathogen *P. syringae* [95]. In a recent preprint report, endogenous CRISPR type III-A system from *Mycobacterium tuberculosis* has been redirected for genome editing, RNA interference, and CRISPRi screening, potentially adding novel tools for the study and control of this important human pathogen [96].

Table 1 shows a comprehensive list of the different published applications of CRISPR-Cas-mediated genome editing in a wide array of bacterial species. As we can see, there have been a recent explosion of CRISPR-Cas methods, often combined with recombineering with variable host-dependent efficiencies. 

## 6. Advantages of Delivering CRISPR-Cas Machinery via Ribonucleoprotein Complexes (RNPs)

**Figure 6:**
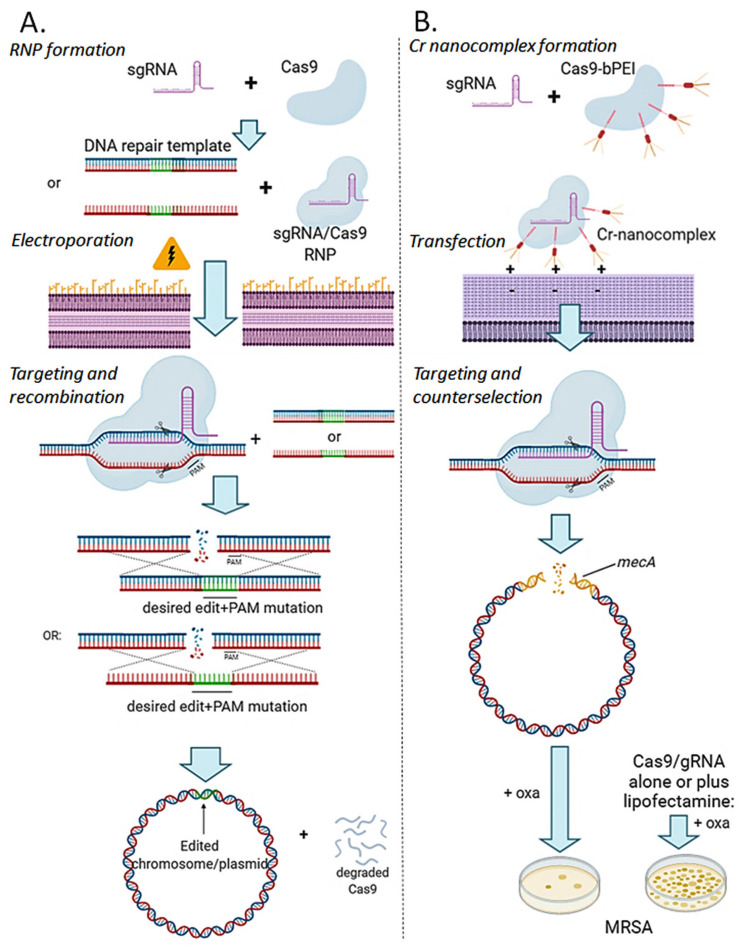
Ribonucleoprotein (RNP) approaches for CRISPR-Cas mediated genome editing. (**A**) RNP electroporation: Recombinant CRISPR nuclease (e.g., Cas9) is combined with in vitro transcribed or synthetic sgRNA to form active Cas9/sgRNA RNP complexes. Electroporation is usually used to form temporary holes in the bacterial cell wall to co-transform the RNPs with a linear single- or double-stranded recombination template harboring the desired edit plus additional mutations at the PAM site to avoid Cas9/sgRNA targeting. Targeting to the desired locus occurs, DNA double-strand break is formed 2–3 bp upstream PAM sequence, which is repaired by double homologous recombination with the linear DNA template. Wild-type allele is replaced by the mutant allele, which is fixed in the target genome or plasmid. Cas9/sgRNA RNPs are maintained only transiently in the cell and are degraded shortly after gene edition. This method does not require the introduction of antibiotic resistance markers or plasmid curing; however, its efficiency would be highly dependent on the transformation amenability and recombination machinery of the bacterial strain. (**B**) Cationic polymer conjugation with Cas9/sgRNA. Recombinant Cas9 is covalently linked to a cationic polymer (bPEI) followed by incubation with sgRNA to form CRISPR nanometric complexes. Electrostatic interactions facilitate binding and incorporation of Cr-nanocomplex into thick-cell walled Gram-positive bacteria. In this example, sgRNA targets incorporated Cas9 to the mecA gene, responsible for methicillin and oxacillin (oxa) resistance in *Staphylococcus aureus* (MRSA). Counterselection of MRSA is efficiently achieved compared to the incubation with RNP alone or combined with the cationic lipid lipofectamine.

Although several alternatives to Cas9 have been recently developed, an alternative mechanism to plasmids that are highly versatile, but depend on the host cell machinery to maintain an efficient, non-toxic expression of the Cas nuclease (but also for Cas nickases or base-editors) and gRNA is the delivery via pre-made Cas9/gRNA RNP complexes. In principle, this approach seems more laborious because of the necessity of purifying active recombinant Cas9 protein from a heterologous system (mostly *E. coli*) and synthesizing gRNA by in vitro transcription. Currently, however, these two elements can also be directly purchased from different vendors. The main advantage of this method is that it does not rely on the host transcription and translation machinery, which also allows to directly evaluate the efficacy of the RNP preparation beforehand by in vitro nuclease assays. Besides, the RNP complex is usually degraded shortly after transfection, avoiding the toxic effects of a continuous Cas9 expression (Figure 6). It also does not require cloning, therefore there is no restriction in the selection of gRNAs that may target a cloning strain genome. It also presents a more concise streamline than the plasmid methods, as no plasmid curing is required (Figure 6A). This strategy has been used to efficiently target and edit eukaryote genomes, e.g., human, mouse, wheat, and zebrafish [97,98,99,100]. SpCas9 is a relatively large protein (160 kDa), which may limit the electroporation efficiency of the nuclease/gRNA complex. 

On the other hand, bacteria with thick cell walls such as Gram-positive bacteria can be very difficult to transfect/electroporate. As an alternative, polymer-derivatized Cas9 has been developed [101]. In this work, direct covalent modification of the protein with a cationic polymer (bPEI) was followed by complexation with a sgRNA to generate nanosized complexes (Figure 6B). Treatment with Cr-nanocomplexes targeting antibiotic resistance inhibited bacterial cell growth on agar plates with oxacillin and demonstrated a higher genome-editing efficiency in methicillin-resistant *Staphylococcus aureus* (MRSA), compared to incubation with SpCas9/sgRNA RNP alone or combined with Lipofectamine, a traditional cationic lipid formulation which showed almost no effect on *S. aureus*. The removal of antibiotic resistance genes through this strategy could prove effective for the control of the rising problem of antibiotic resistance, while maintaining commensal bacteria in microbiota. Additionally, novel lipid nanoparticle formulations such as SORT (selective organ targeting) for Cas9 mRNA and sgRNA [102], and polyethylene glycol phospholipid-modified cationic LNP for Cas9/sgRNA plasmid [103] have shown a high efficiency in mammalian cells. However, it remains to be evaluated if they can also be redirected for genome editing in bacteria.

**Figure 7:**
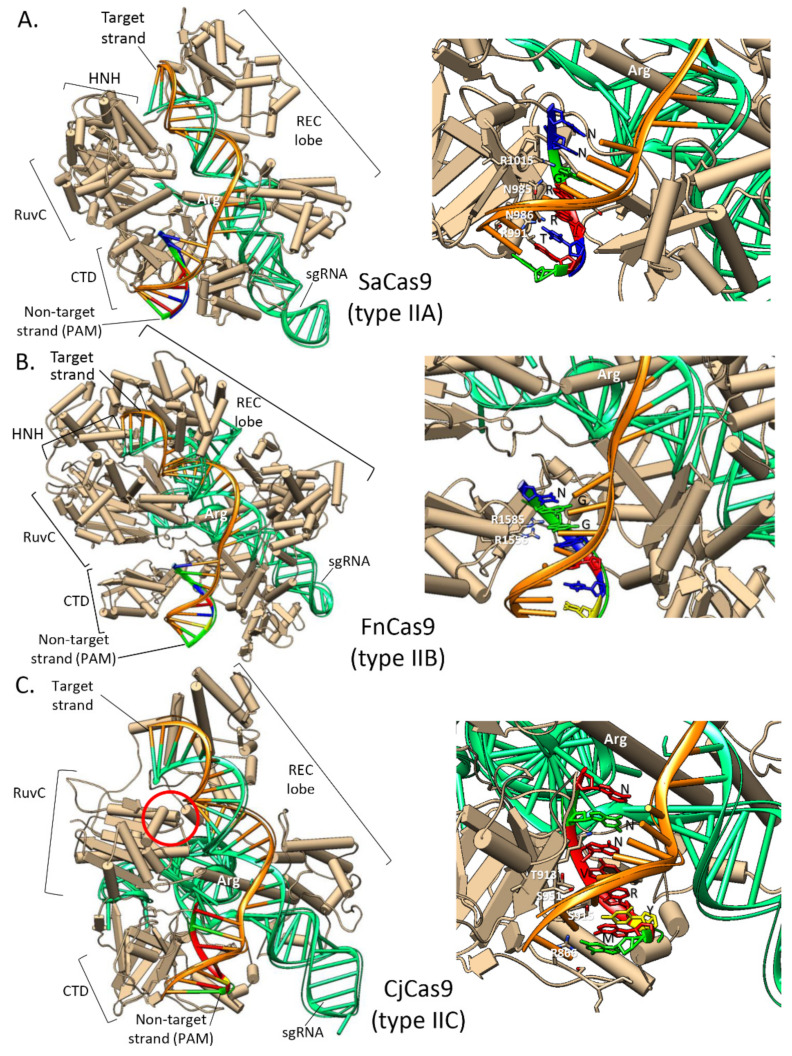
Structures of Cas9 orthologs reveal conserved and divergent features among CRISPR–Cas9 systems (subtypes IIa-IIb-IIc). (**A**) (**Left**) Crystal Structure of the SaCas9–sgRNA–target DNA complex (PDB ID 5CZZ): (**right**) base-specific contacts between CTD domain and PAM nucleotides (NNGR(A or G)R(A or G)T). (**B**) (**Left**) Structure of FnCas9–sgRNA–target DNA complex (PDB ID 5B2O); (**right**) PAM (NGG) recognition by arginine residues of FnCas9 CTD domain. (**C**) (**Left**) Structure of CjCas9–sgRNA–target DNA complex (PDB ID 5X2H); (**right**) base-specific contacts between the CTD domain and PAM nucleotides (NNNV(A or C or G)R(A or G)Y(C or T)M(A or C) in this structure; optimal in vivo PAM has been determined as NNNNRYAC by [107]). The HNH domain has been deleted for crystallization, the red circle indicates its expected position in the CjCas9 structure [110]. PDB structures were drawn with UCSF Chimera v.1.14.

As an alternative to SpCas9 there are several Cas9 orthologs whose structures and mechanisms have also been characterized, which present similar domain architecture, although the sequence homology and length can vary greatly (∼900–1600 amino acid residues). Type II CRISPR nucleases such as Cas9 orthologs can be classified in three subgroups depending on their Cas operon architecture: IIA (cas9, cas1, cas2, cas4), IIB (cas9, cas1, cas2, Csn2), and IIC (cas9, cas1, and cas2 only) [104]. In Figure 7 we show the crystal structure of some representatives from each subgroup. Structural comparisons reveal a relatively conserved catalytic core and a highly conserved arginine-rich bridge helix essential for R loop formation (DNA unwinding) and subsequent DNA cleavage [105]. There is also a less conserved alpha-helical REC lobe essential for guide RNA binding and a divergent CTD that is responsible for both the PAM recognition and the guide RNA repeat–antirepeat heteroduplex binding [104]. The divergent CTD domain may explain the differences in the PAM recognition sequence specific for each Cas9 ortholog (Figure 7). Despite this, the use of smaller Cas9 orthologs is highly valuable to ameliorate issues regarding large SpCas9 packing into vectors and difficulty to transform. For example, in *Trypanosoma cruzi*, because of a highly complex plasma-membrane glycocalyx, electroporation of large SpCas9/gRNA RNPs is not feasible; this issue has been addressed by using the smaller Cas9 ortholog from *Staphyloccocus aureus* Cas9 (SaCas9, 123 kDa), with optimal results for gene knock-outs, gene deletions, and endogenous gene-tagging [106]. Other alternative Cas9 orthologs that have been used for genome editing in eukaryotes are those from *Campylobacter jejunii* (CjCas9, 116 kDa) [107], *Neisseria meningitides* (NmCas9, 124 kDa) [108], and *Streptococcus thermophilus* (St1Cas9, 129 Kda and St3Cas9, 161 kDa) [109] with more complex PAM sequence requirements. In the case of NmCas9 (PAM: NNNNGATT), a mismatch and indels study found an overall improvement over SpCas9 [108]. This indicates that a rare PAM sequence limits the number of off-targets for any given gRNA, providing a more specific genome-editing tool.

## 7. Editing of Fluorescent-Protein Genes to Measure Efficiency of CRISPR-Cas9 in Prokaryotes 

Feasibility of the CRISPR-Cas system among prokaryotes varies greatly depending on several factors, e.g., Cas proteins cytotoxicity, AT genome content, and available genetic transformation methods. In this regard, evaluation of loss of fluorescence in GFP-expressing bacteria serves as a straightforward way to assess CRISPR-Cas activity in vivo in different species. This has been applied in *E. coli* with a dual-plasmid system, one encoding for Cas9 and GFP-specific gRNA expression, and another for GFP expression. GFP-plasmid loss varied between 80% and 98% of colonies depending on the gRNA sequence [111]. The system can also be used to assess the efficiency of CRISPR-Cas-mediated gene editing. It has been shown that the Tyr66-His mutant (encoded by the single base substitution 196T>C) shifts wild-type GFP absorption and emission toward the blue spectrum, thus creating blue fluorescent protein (BFP) [112]. A GFP to BFP conversion assay has recently been applied to evaluate a plasmid-based CRISPR/Cas9 system in *Methylococcus capsulatus* [113].

## 8. Discussion

CRISPR-Cas technology has revolutionized the genome editing and has become the state-of the-art approach in eukaryotic organisms. Its application in prokaryotes has been slower but is quickly being adapted to several bacteria of industrial and biomedical importance. Several challenges need to be addressed for a widespread application of this revolutionary technology in bacteria (Table 2). 

In particular, if it is to be superior to current methods based on suicide plasmids and recombineering that keep being adapted and improved in bacteria [114], efficiency needs to be adjusted regarding available transformation tools and genetic accessibility for each species. The greatest disadvantage of CRISPR-editing tools in bacteria is so far the cytotoxicity induced by a continuous expression of foreign CRISPR-nucleases. In fact, CRISPR-Cas9 is mostly utilized as a counter-selection mechanism against colonies that do not undergo a desired genomic edit, rather than as an actual genome editing tool in bacteria. Several alternatives have been developed including inducible-promoters, nCas9, dCas9, Cas12a and base-editors with different degrees of success depending on the bacterial strain or species. A complex chimeric effector combining engineered dCas9 without PAM binding activity coupled to other inducible DNA-binding protein such as PhlF domain has also been developed, with reduced toxicity in *E. coli* [115]. Additionally, the development of highly efficient prime-editors that do not require a DNA repair template or DSB still needs to be explored in bacteria. 

However, the continuous expression of any foreign protein with DNA-binding/editing activity seems to be particularly toxic for many bacteria. The natural function of CRISPR as an adaptive immune system is highly controlled in prokaryotes. Ultimately, more research to fully understand and being able to harness endogenous CRISPR loci (spacers and Cas proteins) for genome editing would be in principle the most effective way to avoid foreign CRISPR systems in bacteria. This approach would apparently require a case-by-case scenario of efficiency and tuning for each native CRISPR effectors. Nevertheless, more recent studies using the native CRISPR machinery (type I or type II) have been reported with high efficiencies [82,83,84,85] (Table 1), indicating that this may be the way to go for biomedical and industrially relevant bacterial species with endogenous CRISPR systems. Continuous research on endogenous CRISPR systems also helps to create and diversify the strategies for heterologous genome editing.

In eukaryotic organisms the ribonucleoprotein (RNP) format with foreign but well characterized Cas enzymes, such as SpCas9, has shown higher efficiency and much lower cytotoxic and off-target effects compared to the plasmids. Further research would show if this strategy could have similar benefits for genome editing in bacteria with the available transformation methods (e.g., electroporation, derivatization with cationic polymers). The RNP approach is by no means limited to SpCas9, as it has been tested successfully, mostly in eukaryotic organisms, with other natural Cas9 orthologs such as SaCas9 and CjCas9 with more complex PAM requirement but smaller and easier to transfect than SpCas9. Also, several Cas9 orthologs have been engineered and promise higher efficiency, specificity, and broader PAM requirements. These novel alternatives expand the available toolbox that should be explored in bacteria to enhance the potential of CRISPR-mediated genome editing in these relevant organisms. 

Ultimately, genome editing would allow the creation of synthetic genomes combining a wide array of genes, metabolic pathways, and even full chromosomes [116] from different organisms to optimize the production of relevant metabolites, e.g., natural products [117]. 

## Figures and Tables

**Table 1:** Strategies for CRISPR-mediated genome editing in bacteria.

Strategies for Editing	Strain	Results	Efficiency	Reference
Scarless Cas9 Assisted Recombineering (no-SCAR) λ-Red	*Escherichia coli MG1655* *pCas9cr4* *pTET promoter*	This method does not leave recombinase recognition site scars, which can cause chromosomal instability and unwanted genomic rearrangements.	85–100%	[57]
Induce a recombinase	*Escherichia coli HME63*	Editing is facilitated by a co-selection of transformable cells and a small induction of recombination in the target site by Cas9 cleavage.	4.8 × 10^5^/5.3 × 10^2^ CFU	[28]
	*Streptococcus* *pneumoniae* *JEN53*	Genome engineering works in highly recombinogenic bacteria.	10^−1^ CFU	[28]
	*Corynebacterium glutamicum*	Enables transformation to be simpler and more convenient than two-plasmid-based CRISPR–Cas9 method.	2.1 × 10^3^ CFU/μg	[34]
	*Lactoccocus lactis*	Is highly efficient, time-saving, and easy-to-use for introducing precise point mutations and performing gene deletion and insertion in a seamless manner.	87%	[35]
	*Lactobacillus plantarum WCFS1*	Combination of RecE/T-assisted HDR and CRISPR–Cas9 targeted chromosomal DSBs offer a general and adaptable strategy to address the low HDR of *Lactobacillus* spp.	>89.4%	[36]
	*Lactobacillus brevis* *ATCC367*		83.3% (⅚ colonies)	[36]
Encode DNA repair template in a plasmid	*Clostridium ljungdahlii*	More rapid, no added antibiotic resistance gene, scar-less and minimal polar effects.	<75%	[41]
	*Lactobacillus plantarum* *NIZO2877*	Uniquely capable of gene insertions. It showed vast differences for Cas9-mediated genome editing between methods and related strains.	10^2^ CFU	[42]
	*Pseudomonas putida* *KT2440*	Adopted for counterselection of the correct mutants.	74.35%	[43]
	*Streptomyces coelicolor*	Improves the genome editing efficiency compared with the currently existing.	60–100%	[44]
	*Staphylococcus aureus* *RN4220*	High editing efficiencies and easy use of a highly efficient transcription-inhibition system.	70–100%	[45]
Inducible promoters	*Escherichia coli*	Introduces various types of genomic modifications with near 100% editing efficiency and to introduce three mutations simultaneously.	83%	[56]
	*Bacillus subtilis*	Shorter time to achieve the mutations. Sometimes it can be very laborious to of the corresponding mutant.	50%	[58]
	*Clostridium acetobutylicum* *ATCC 824*	Two-plasmid inducible CRISPR/Cas9 genome editing tool was successfully developed. This method enables the rapid introduction of marker-free genomic modification of any type, from the substitution of a few nucleotides to large deletions or insertions.	10^−3^ CFU/total colonies	[59]
	*Lactococcus lactis* *dCas9*	CRISPRi, is used in conjunction with a nisin-inducible promoter, for non-toxic, precise, targeted genome regulation and represents a valid alternative to RNAi.	50-fold mRNA downregulation	[60]
Nucleases of CRISPR-like DNA Nickase	*Corynebacterium glutamicum*	Using either two plasmids or one-plasmid consisting of FnCpf1, CRISPR RNA, and homologous arms.	86–100% for small changes	[10]
	*Francisella novicida*	CRISPR arrays are processed into mature crRNAs without the requirement of an additional trans-activating crRNA (tracrRNA) Cpf1-crRNA complexes efficiently cleave target DNA proceeded by a short T-rich protospacer-adjacent motif (PAM), in contrast to the G-rich PAM Cpf1 introduces a staggered DNA double-stranded break with a 4 or 5-nt 5′overhang.	25–100% in HEK293FT	[69]
	*Mycobacterium smegmatis*	CRISPR-Cas12a can efficiently introduce point mutations into PAM- and crRNA-targeting regions.	80%	[30]
	*Yersinia pestis KIM 6+*	CRISPR-Cas12a as a useful method for genetic manipulation of chromosomal and plasmid DNA.	81–83%	
Base editors (cytidine deaminase)	*Escherichia coli*	Use of uracil DNA glycosylase inhibitor in combination with a degradation tag (LVA tag) resulted in a robustly high mutation efficiency, which allowed simultaneous multiplex editing of six different genes.	61.7–95.1%	[74]
	*Klebsiella pneumonia*	Development of a cytidine base-editing system, pBECKP, for precise C → T conversion by engineering the fusion of the cytidine deaminase APOBEC1 and a Cas9 nickase.	25–100%	[32]
	*Pseudomona aeruginosa*	Development of a genome editing method pCasPA/pACRISPR by harnessing the CRISPR/Cas9 and the phage λ-Red recombination systems. The method allows for efficient and scarless genetic manipulation.	93–100%	[75]
Base editors (adenine deaminase)	*Rhodobacter sphareroides*	CBEs (cytosine base editors) and ABEs (adenine base editors) serve as alternative methods for genetic manipulation of bacteria that are hard to be directly edited by Cas9-sgRNA.	43–97%	[76]
	*Staphylococcus aureus*	This method substantially simplifies the genome editing process and achieves the conversion of adenine to guanine via an enzymatic deamination reaction and a subsequent DNA replication process rather than HDR.	50–100%	[77]
Endogenous CRISPR systems	*Clostridium difficile* *630Δerm* *R20291*	Repurposing of endogenous Type IB CRISPR system coupled to a CRISPR mini-array plasmid to cause DSB-induced auto-immunity and also to generate *Δhfq* mutant strain with plasmid-encoded homologous repair DNA template.	30% to 100%	[93]
	*Heliobacterium modesticaldum*	Redeployment of endogenous type IA CRISPR system, coupled to a homologous recombination plasmid carrying a miniature CRISPR array, which targets sequences in pshA (downstream of a naturally occurring PAM sequence) produced non-phototrophic transformants with clean replacements of the pshA gene.	80%	[94]
	*Mycoplasma gallisepticum* *S6*	Using of endogenous MgaCas9 coupled to three constructs carrying different CRISPR arrays targeting regions in the ksgA gene. This leads to NHEJ-induced mutations (insertions and deletions) that prevent ribosomal methylation, which in turn confers resistance to the aminoglycoside antimicrobial kasugamycin, enabling selection of mutants.	1.18 × 10^6^ vs. 2.47 × 10^8^ CCU/mL (3 days cultures with vs. without kasugamycin) 63–100% indel ocurrence	[89]
	*Pseudomonas aeruginosa*	Repurposing and optimization of endogenous Type I CRISPR system (PaeCas3c) for genome engineering with a single crRNA and selecting only for survival after editing via native A-EJ. Self-targeting crRNAs leads to large genomic deletions (7–424 kb). When provided with a HDR template PaeCas3c promotes recombination compared to SpCas9.	A-EJ: 20–40% of surviving colonies with native crRNAs 94–100% with modified-repeat crRNAs HDR: 22% for 249 kb deletion (vs. 0% for SpCas9) 61% for 56 kb deletion (vs. 11% for SpCas9) 100% for 0.17 kb (vs. 78% for SpCas9).	[95]

**Table 2:** Advantages and disadvantages of most commonly used genome-editing methodologies in bacteria.

Method	Advantages	Disadvantages
“Suicide” plasmids	- Low cost - Does not require specialized strains - Useful for large genomic deletions or targeted gene disruption	- Low efficiency - High rate of false positives - Often requires several rounds of antibiotic selection - Long homology flanking regions (~1 Kb) to the desired edit need to be cloned
“Recombineering” (Lambda Red, RecE/T)	- Low cost - Highly efficient, particularly for small-scale edits - Utilizes DNA templates with only short regions of homology (50 bp) to promote gene edition by homologous recombination	- Requires development of specialized strains with controlled foreign recombinase expression. - Usually requires counter-selection steps to eliminate antibiotic resistance markers from the genome
ClosTron method (Retrotransposition-Activated Marker)	- Can be programmed by designing a 344 bp region homologous to the target gene - Broad-host range of Ll.LtrB intron theoretically allows its use in any bacterial species	- So far only tested in members of the Clostridium (Clostridiodes) genus - Requires extensive cloning or expensive out-sourced synthesis of modified targeting intron - Application of the method is straightforward only for targeted gene disruption
CRISPR-Cas (plasmid-encoded)	- Low cost - Can be combined with recombineering for an enhanced efficiency - Highly customizable - Double strand breaks induce cell death in non-edited cells diminishing background (false positive colonies) - Highly versatile genome editing from large genome deletions/insertions to single base mutations.	- High cytotoxicity of Cas9 expression can alter morphology and survival even when devoid of nuclease activity due to steric hindrance posed by Cas9 PAM binding and subsequent DNA unwinding activity along the genome. - Induction of off-target effects (undesired genome edits) due to non-specific DNA cleaving, particularly after prolonged Cas9/gRNA expression
CRISPR-Cas (Endogenous systems)	- Do not necessitate the expression of a foreign CRISPR nuclease - Highly programmable by altering the homology repair template and the CRISPR array sequence	- Requires extensive characterization of the endogenous CRISPR system (nucleases, PAM requirement, efficiency, etc.) and DNA repair pathways (e.g., NHEJ) for each particular species/strain
